# Value of attenuation correction in stress-only myocardial perfusion imaging using CZT-SPECT

**DOI:** 10.1007/s12350-015-0374-2

**Published:** 2016-01-15

**Authors:** J. D. van Dijk, M. Mouden, J. P. Ottervanger, J. A. van Dalen, S. Knollema, C. H. Slump, P. L. Jager

**Affiliations:** 1 Department of Nuclear Medicine, Isala Hospital, PO Box 10400, 8000 GK Zwolle, The Netherlands; 2 Department of Cardiology, Isala Hospital, Zwolle, The Netherlands; 3 Department of Medical Physics, Isala Hospital, Zwolle, The Netherlands; 4 MIRA: Institute for Biomedical Technology and Technical Medicine, University of Twente, Enschede, The Netherlands

**Keywords:** Attenuation correction, stress-only, myocardial perfusion imaging: SPECT, CdZnTe

## Abstract

**Background:**

Attenuation correction (AC) improves the diagnostic outcome of stress-only myocardial perfusion imaging (MPI) using conventional SPECT. Our aim was to determine the value of AC using a cadmium zinc telluride-based (CZT)-SPECT camera.

**Methods and results:**

We retrospectively included 107 consecutive patients who underwent stress-optional rest MPI CZT-SPECT/CT. Next, we created three types of images for each patient; (1) only displaying reconstructed data without the CT-based AC (NC), (2) only displaying AC, and (3) with both NC and AC (NC + AC). Next, two experienced physicians visually interpreted these 321 randomized images as normal, equivocal, or abnormal. Image outcome was compared with all hard events over a mean follow-up time of 47.7 ± 9.8 months. The percentage of images interpreted as normal increased from 45% using the NC images to 72% using AC and to 67% using NC + AC images (*P* < .001). Hard event hazard ratios for images interpreted as normal were not different between using NC and AC (1.01, *P* = .99), or NC and NC + AC images (0.97, *P* = .97).

**Conclusions:**

AC lowers the need for additional rest imaging in stress-first MPI using CZT-SPECT, while long-term patient outcome remained identical. Use of AC reduces the need for additional rest imaging, decreasing the mean effective dose by up to 1.2 mSv.

## Introduction

Myocardial perfusion imaging (MPI) using single photon emission computed tomography (SPECT) is a well validated and frequently used non-invasive method in the evaluation of known or suspected coronary artery disease (CAD).^1^,^2^ Stress-only MPI is recommended by both European and American guidelines in appropriately selected patients to reduce radiation dose and improve laboratory efficiency.^3^,^4^ However, attenuation artifacts are common and result in a higher necessity for additional rest imaging and in a lower diagnostic accuracy.^4^ Additional use of attenuation correction (AC) improves the specificity and reduces the necessity for additional rest imaging,^5^-^8^ and is therefore recommend by the international guidelines.^3^,^4^

The recently introduced ultrafast cardiac SPECT cameras with cadmium zinc telluride-based (CZT) detectors provide superior image quality, resulting in shorter acquisition times, lower radiation doses, and less equivocal scans, facilitating stress-only imaging.^9^,^10^ However, it is unknown whether this superior image quality obviates the use of AC for such cameras. The aim of this study was therefore to determine the added value of AC in stress-only MPI using a CZT-SPECT camera.

## Methods

### Study Population

We retrospectively included 107 consecutive low to intermediate risk patients with suspected CAD referred to our hospital for a clinically indicated CZT-SPECT stress MPI including CT-based attenuation correction (Discovery NM/CT 570c, GE Healthcare).^11^ The pretest likelihood of CAD was assigned according to the criteria of Diamond and Forrester, with a risk threshold of <13.4% for low risk, between 13.4% and 87.2% for intermediate risk, and >87.2% for high risk.^12^ All patients were scanned in July and August 2010 allowing to obtain a long-term follow-up. All patients provided written informed consent for the use of their data for research purposes.

### Clinical Information

At the time of examination, all patients completed a questionnaire regarding demographic information, prior medical history, cardiac risk factors, and current medication use. These data were verified and complemented with demographic and clinical information collected from medical records.

### Patient Preparation and Image Acquisition

Patients were instructed to refrain from caffeine-containing beverages for 24 h. Pharmacological stress was induced by intravenous adenosine (140 μg/kg min for 6 minutes) or dobutamine (10 μg/kg min increased to a maximum of 50 μg/kg min until 85% of the predicted maximum heart rate was reached). Only pharmacologic stress was used due to logistic reasons, in particular the high patient throughput in our center.^9^ Patients were injected intravenously at peak stress with a fixed dose of 370 MBq (10 mCi) Tc-99 m tetrofosmin (500 MBq (13.5 mCi) for patients with a body weight of more than 100 kg). When optional rest imaging was clinically indicated, patients received 3 hours after the stress activity injection intravenously a fixed dose of 740 MBq (20 mCi) Tc-99 m tetrofosmin (1000 MBq (27 mCi) for patients >100 kg).

ECG-gated SPECT acquisition was performed 60 minutes post-injection according to the guidelines with the patient in supine position with arms placed above their heads.^3^,^13^ Prior to scanning, the patient’s heart was positioned in the center of the CZT-SPECT scanner using real-time persistence imaging. Scans were acquired during 5 minutes using a 20% symmetrical energy window centered at 140 keV. Post SPECT acquisition, an unenhanced low-dose CT scan during a breath-hold was made to provide the attention map of the chest. This scan was made using a 5.0-mm slice thickness, 800 ms rotation time, pitch of 1.0, collimation 64 × 0.625 mm, tube voltage of 120 kV, tube current of 20 mA, and an irradiated body length of 24.4 cm. The dedicated heart CZT-SPECT system that we used has been described repeatedly in the literature.^9^,^10^,^14^,^15^ In short, the scanner uses 19 pinhole detectors centered around the myocardium each containing 32 × 32 pixelated (2.46 × 2.46 mm^2^) high-sensitive CZT-elements.

**Figure 1:**
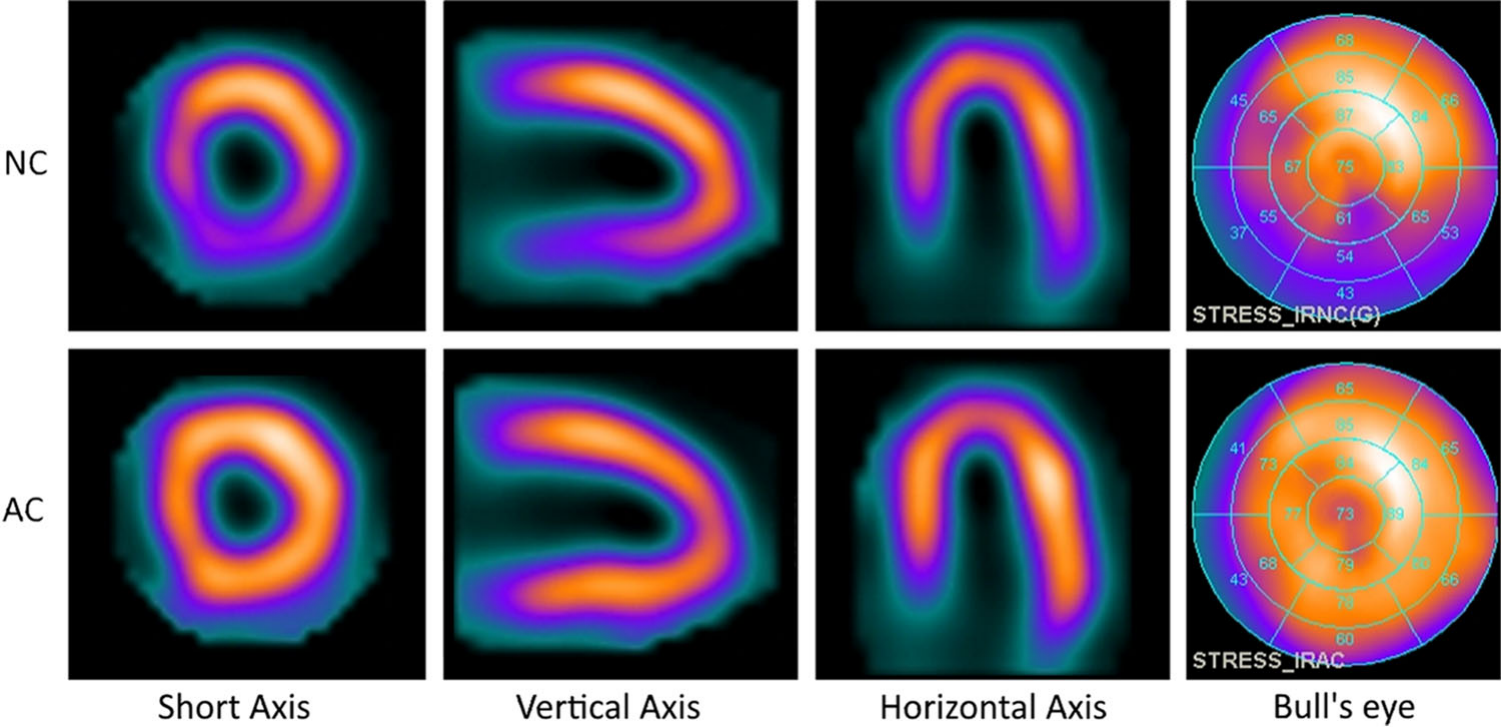
Key images from a stress MPI CZT-SPECT scan reconstructed without (*top row*) and with attenuation correction (*bottom row*), where a possible inferior defect in the non-corrected images is corrected using attenuation correction. The images are from a typical patient (72 year-old male, 67 kg, BMI 22 kg/m^2^). Corresponding short, vertical, horizontal axis and a parametric bull’s eye plot are shown from left to right

SPECT data were reconstructed without and with CT-based AC by applying an iterative dedicated reconstruction algorithm with maximum-likelihood expectation maximization for both NC and AC scans (Xeleris software, GE Healthcare). Next, the scans were displayed in the traditional short, vertical long, and horizontal long axes, as illustrated in Figure 1.

### Image Interpretation

Three types of image sets were created for interpretation from each MPI stress acquisition; (1) reconstructed data without AC (NC), (2) with AC only, and (3) with both NC and AC (NC + AC). Next, two experienced readers interpreted in consensus the total of 321 randomized and blinded images as normal; no evidence of perfusion deficits, equivocal; possible perfusion deficits, or abnormal; most likely perfusion deficits. All images interpreted as equivocal or abnormal were considered to require rest imaging in this study, in accordance with international guidelines.^3^,^4^ Readers were unaware of the patients’ history or other clinical findings. In addition, the mean radiation dose was calculated for the NC and AC stress-only approach, correcting for the possible decrease in the percentage of rest scans required. To estimate the radiation dose, an effective dose conversion factor of 6.9 × 10^−3^ mSv/MBq was used for Tc-99 m tetrofosmin and a thorax conversion factor of 0.017 mSv/mGy cm for the CT-scans.^16^,^17^

### Clinical Follow-Up

We recorded follow-up information of all patients by reviewing hospital records, performing scripted telephone interviews with patients and by contacting general practitioners. The interval between the MPI acquisition and the date of the latest consultation or examination was used to determine follow-up length. Two follow-up endpoints were defined, (1) the occurrence of hard events, defined as all-cause death or non-fatal myocardial infarction and (2) occurrence of hard cardiac events, defined as cardiac or unknown death or non-fatal myocardial infarction. Non-fatal myocardial infarction was defined based on the criteria of typical chest pain, elevated cardiac enzyme levels, and typical changes on the ECG as defined by Thygesen et al.^18^ Data were censored at the first cardiac event.

### Statistical Analysis

Baseline characteristics were analyzed using Stata (StataSE 12.0) and expressed as mean ± standard deviation (SD). Pretest likelihood was determined using the updated clinical prediction model by Genders et al.^19^ The diagnostic confidence, defined as the percentage of definite MPI interpretations (either normal or abnormal), was compared between the three image types (NC, AC, or NC + AC) using the Cochran’s Q test. The same test was used to compare the number of images interpreted as equivocal or abnormal, where rest imaging was considered necessary, between the three image types (NC, AC, or NC + AC). The influence of gender on the diagnostic confidence and necessity for rest imaging was tested for each image type using the Fisher’s exact test. The influence of BMI on both these outcomes was tested using an unpaired *t* test for all three image types.

The hazard ratios for using only AC or NC + AC instead of NC were calculated for the images interpreted as normal and compared using the Cox-regression model with a shared frailty to account for the paired data. In addition, the annualized event rates were calculated and compared between the patients for whom the scans were interpreted as normal and scans which were interpreted as equivocal or abnormal for the three image types using the Cox-regression model.

The level of statistical significance was set to 0.05 (two-sided) for all statistical analyses.

## Results

The baseline characteristics are summarized in Table 1.

**Table 1:** Baseline characteristics of all 107 patients with suspected CAD referred for CZT-SPECT imaging

Characteristic	
Age (years)	60.2 ± 12.4
Male gender (%)	43.0
Body weight (kg)	83.8 ± 16.0
BMI (kg/m^2^)	28.1 ± 4.7
Diabetes (%)	9.3
Hypercholesterolemia (%)	43.0
Hypertension (%)	64.5
Current smoking (%)	23.4
Family history of CAD (%)	36.4
Adenosine induced stress (%)	96.0
Pretest likelihood (%)	37.7

Data are presented as percentages or mean ± SD

### Image Interpretation

**Figure 2:**
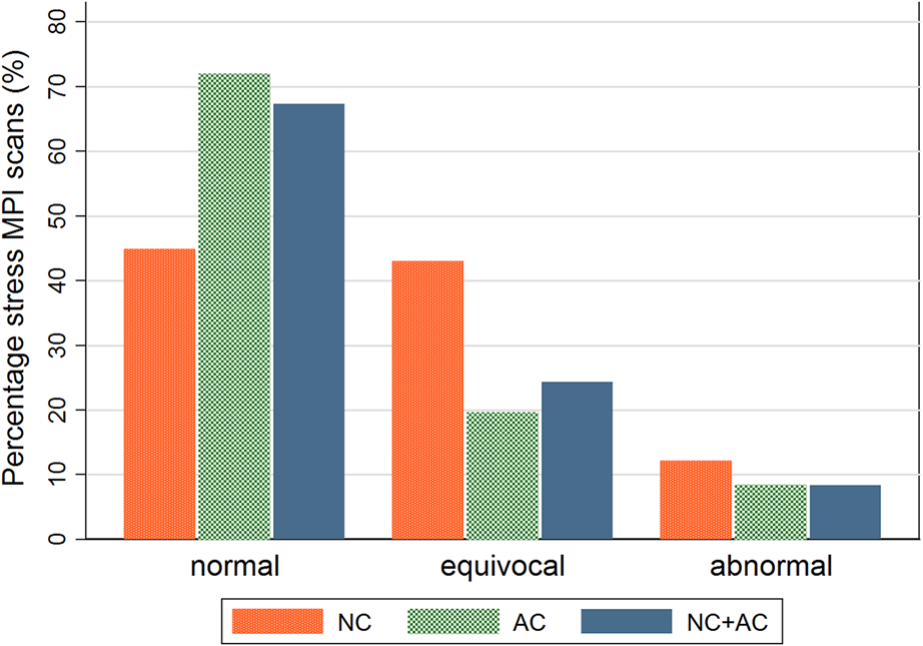
*Bar graph* showing the scan outcomes (normal, equivocal, or abnormal) for all 107 stress MPI studies using only non-attenuation corrected (NC), only attenuation corrected (AC), or both NC and AC images (NC + AC). The number of equivocal and abnormal interpreted scans and, hence, the perceived necessity for rest imaging were higher when only using the NC images (*P* < .001)

The impact of attenuation correction on the interpretation of the stress-only scans is illustrated in Figure 2. The percentage of scans interpreted as normal, indicating the percentage of stress-only scans, increased from 45% (*n* = 48) for the NC images to 72% (*n* = 77) using AC only and to 67% (*n* = 72) using the NC + AC images (*P* < .001). In addition, the diagnostic confidence, scans interpreted as either normal or abnormal, increased from 57% using the NC images, to 80% when using only the AC images, and to 76% using the NC + AC images, (*P* < .001). No influence of gender or BMI was observed for all three image types for the need for rest imaging (*P* > .07 and *P* > .21, respectively) or diagnostic confidence (both *P* > .11).

The mean radiation doses for stress MPI, rest MPI, and the unenhanced CT-scan were 2.7, 5.4, and 0.29 mSv, respectively. Without applying AC, 55% of the patients would have underwent rest imaging, resulting in a mean effective dose of 5.7 mSv. When applying AC on the stress images, 28% or 33% of the patients would have underwent additional rest imaging—depending on the use of AC or NC + AC images—reducing the mean effective dose to 4.5 or 4.8 mSv including the additional AC CT-scan.

### Clinical Follow-Up

Follow-up was obtained for all patients. The mean follow-up duration was 47.7 ± 9.8 months (median 51.8 months, interquartile range 45.5-53.8 months). During follow-up, one patient experienced a non-fatal acute myocardial infarction requiring a primary percutaneous intervention and seven patients (6.5%) died during follow-up. One died from a cardiac cause, one from an unknown cause, and the other five from non-cardiac causes.

**Figure 3:**
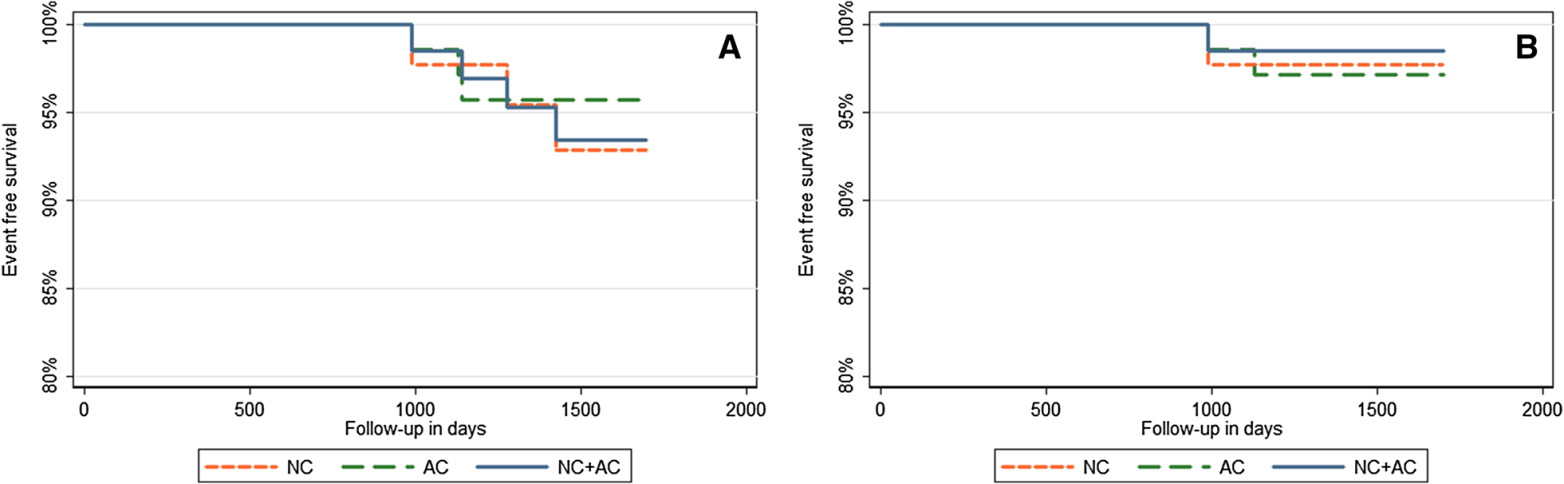
Kaplan-Meier curves of event-free survival of (**A**) all-cause death or non-fatal myocardial infarction and (**B**) cardiac or unknown death or non-fatal myocardial infarction, based on the three different image types: Non-attenuation corrected (NC), only attenuation corrected (AC) or both NC and AC images

Despite the higher number of scans interpreted as normal when using the AC only or NC + AC images as compared to the NC images, the hazard ratios were not statistically different between these three groups (*P* > .93). The hazard ratios for the normally interpreted images varied for the hard events between 0.97 and 0.99 and for the cardiac hard events between 0.88 and 1.01 for the three image types, as shown in Figure 3.

The annualized hard event rates for the NC images did not differ between the images interpreted as normal (1.60%) and equivocal or abnormal (2.20%, *P* = .30), as shown in Table 2. These event rates did also not differ between the images interpreted as normal (1.40%) and equivocal or abnormal (2.80%) for the NC + AC images (*P* = .30). However, the annualized hard event rate was significantly lower in patients in whom the scans were interpreted as normal (0.97%) as compared to patients with equivocal or abnormal stress SPECT results (4.29%) using the AC images (*P* = .04). The annualized cardiac hard event rates varied between 0.35% and 0.65% for the normally interpreted images and between 0.86% and 1.44% for images interpreted as equivocal or abnormal and did not differ for the NC, AC, and NC + AC images between the images interpreted as normal or as equivocal or abnormal (*P* > .23).

**Table 2:** Hard event and hard cardiac event rates for the three image types (NC, AC, or NC + AC) for scans interpreted as normal and as equivocal or abnormal, including the 95% confidence intervals

		NC		AC		NC + AC
All-cause mortality or myocardial infarction						
Normal	1.60%	(0.5%–4.8%)	0.97%	(0.31%–3.0%)	1.40%	(0.5%–3.8%)
Equivocal or abnormal	2.20%	(0.9%–5.2%)	4.29%	(1.8%–10.3%)	2.80%	(1.1%–7.5%)
Cardiac or unknown mortality or myocardial infarction						
Normal	0.50%	(0.07%–3.7%)	0.65%	(0.2%–2.6%)	0.35%	(0.05%–2.5%)
Abnormal	0.86%	(0.22%–3.5%)	0.86%	(0.12%–6.1%)	1.44%	(0.3%–5.8%)

The annualized event rates for the scans interpreted as normal did not differ between the three different image types for both hard events and hard cardiac events (*P* > .97 and *P* > .88, respectively)

## Discussion

In this study, we have demonstrated the value of AC in stress CZT-SPECT interpretation. The use of AC in CZT-SPECT imaging increased the certainty of interpretation with more scans interpreted as normal and less equivocal scans that could improve stress-only imaging without compromising its prognostic value as now demonstrated by our results.

Since the introduction of cardiac CZT gamma cameras with stationary multi-pinhole collimators, several studies have evaluated various imaging protocols with regard to acquisition time, image quality, radiation dose, and diagnostic accuracy.^9^,^10^,^20^-^23^ However, studies assessing the clinical value of AC in CZT-SPECT imaging are limited and have not yet been studied in stress-only protocols and prognostic studies.^24^ We compared the added value of AC for stress-only imaging with CZT SPECT in stable patients with a suspicion of CAD. In addition, we also studied the incidence of hard events to assess the prognostic implications of AC. We found a significant improvement in diagnostic confidence with less equivocal findings enhancing stress-only imaging. More importantly, the incidence of events in patients with normal stress-only findings based on attenuation corrected images did not exceed that encountered in patients with normal stress SPECT findings based on non-corrected SPECT findings.

Our results correspond well with previous studies using conventional SPECT cameras reporting reductions in the necessity for rest imaging varying between 17 and 48%.^5^-^8^ Yet, the necessity for rest imaging varied between those studies which is most likely due to different study designs and populations. Heller et al reported a reduced necessity for additional rest imaging from 77 to 43%,^6^ which is in agreement with the study by Trägårdh et al showing a decrease from 49% to 32% when using AC.^7^ The latter study shows similar results to our study, although we used a more sensitive CZT-based camera associated with a lower need for rest imaging with similar follow-up.^9^ However, due to the lack of prognostic data in the study by Trägårdh et al, the prognostic value of using solely stress-only imaging in this relatively large group is unknown. Moreover, Mathur et al, reported an even higher reduction in the need for rest imaging from 58% to 10% when using AC.^8^ They reported similar or slightly higher annualized hard cardiac event rates for patients with scans interpreted as normal as compared to patients with abnormal scans. However, the scans of only two of the 14 patients who encountered a hard cardiac event in the follow-up period were interpreted as abnormal in their study, possibly indicating a loss in sensitivity. Although the possible increase of false negative scans is one of the major concerns when solely using AC images, several large studies have reported an increase in prognostic value when using AC.^25^-^28^ This is in line with the present study, showing a comparable hazard ratio for the occurrence of cardiac events when the scans were interpreted as normal when using AC.

The use of AC resulted in a not significantly lower number of equivocal scans and a lower, but also not statistically different, hazard ratio for the normally interpreted scans in comparison to using both AC + NC images. Due to possible errors introduced by the manual alignment of the AC map to the NC images and to distinguish overcorrection by AC, we recommend to use AC images in combination with NC images in routine clinical practice, as suggested by current guidelines.^3^,^4^

Several assumptions were made in this study. First, no clinical information, i.e., ECG, gender, or age, were available when interpreting the images to ensure proper blinding and to prevent additional influences, which could have limited the generalizability. Yet, the necessity for rest imaging using NC + AC in the present study (33%) was found to be similar to a previous retrospective clinical study in our center (35%) using a comparable patient population. Hence, the influence of the absence of clinical information was considered to be limited.^9^ Second, the influence of ECG-gating on the interpreter confidence and perceived necessity for rest imaging was not taken into account in this study. However, ECG-gated acquisition is already applied routinely, and the influence was considered to be limited, as previously demonstrated by Heller et al.^6^ Third, the differences in hazard ratios were compared between the three image types using follow-up data of a relatively small group, while diagnostic outcomes were not assessed. However, assessing the diagnostic accuracy was beyond the scope of our study. In addition, this study may have been underpowered for prognostic outcome comparisons. Finally, the additional use of prone imaging in addition to AC was not taken into account in this study. Yet, Malkerner et al reported a higher decrease in the number of equivocal scans when using supine imaging with AC as compared to using both prone and supine imaging without AC.^29^ Moreover, using prone imaging with AC in addition to supine imaging with AC did not reveal any improved results, and therefore, they suggested that additional prone imaging can be used as an alternative when AC is not available. In addition, one should realize that although AC improves the scanner capacity due to the lower number of rest examinations necessary, this improved laboratory efficiency is partly compromised by the longer time patients lie in the scanner. Moreover, the additional post-processing activities required for AC do also increase the workload for the technologist, taking up to 5 minutes extra per patient.

## Conclusion

The use of attenuation correction in stress-only imaging using a CZT-based SPECT camera generates more normal and fewer equivocal scans and therefore increases diagnostic confidence. Although we did not assess the diagnostic accuracy, long-term patient outcome was identical between NC- and AC-based interpretations. Use of AC reduces the need for additional rest imaging and, hence, helps in decreasing the mean effective dose and improve laboratory efficiency.

## New Knowledge Gained

The need for rest imaging after stress-first MPI using CZT-SPECT can be lowered when using CT-based attenuation correction while maintaining the high diagnostic accuracy. Hence, this study shows that the findings of previous studies—demonstrating the beneficial effect of applying attenuation correction in MPI using conventional SPECT cameras—seem to hold for CZT-based SPECT cameras.

## Abbreviations

AC: Attenuation correction
CZT: Cadmium zinc telluride-based
MPI: Myocardial perfusion imaging
NC: Non-attenuation corrected
NC + AC: Non-attenuation corrected and attenuation corrected
SPECT: Single photon emission computed tomography
